# Survey data on factors affecting negotiation of professional fees between Estate Valuers and their clients when the mortgage is financed by bank loan: A case study of mortgage valuations in Ikeja, Lagos State, Nigeria

**DOI:** 10.1016/j.dib.2017.04.047

**Published:** 2017-05-01

**Authors:** Chukwuemeka O. Iroham, Hilary I. Okagbue, Olalekan A. Ogunkoya, James D. Owolabi

**Affiliations:** a Department of Estate Management, Covenant University, Ota, Nigeria; b Department of Mathematics, Covenant University, Ota, Nigeria; c Department of Building Technology, Covenant University, Ota, Nigeria

**Keywords:** Mortgage valuation, Bank loan, Questionnaire, Estate Valuers, Clients, Ikeja, Nigeria

## Abstract

In this article, two sets of questionnaires were administered to professionals and clients (commercial banks) on their willingness to negotiate the professional fees charged by the Estate Valuers assuming that the mortgage in valuation was financed by bank loan. A range of fees options were provided. Other factors such as the business environment and mortgage valuation can influence the negotiated fees when the data obtained from the survey data is analyzed.

**Specifications Table**

**Table:** 

Subject area	Economics
More specific subject area	Mortgage Valuation.
Type of data	Tables and Text files
How data was acquired	Field survey
Data format	Raw
Experimental factors	Simple random sampling of Estate Valuers and their clients in Ikeja, Nigeria
Experimental features	Sample selection of views of clients and professionals on negotiated fees payable or receivable by each party as appropriate
Data source location	Nigeria.
Data accessibility	All the data are in this data article

**Value of the data**

• Can be used for educational and research purposes and by mortgage industry.
• The data can provide insight on the factors responsible for the professional fees paid by clients for mortgage valuation when the properties are acquired through bank loans.
• The questionnaires can be adapted, adopted or modified for a similar research.
• The data is valuable for socioeconomic analysis of mortgage valuation and ethics in negotiation of professional fees. See [1], [2], [3], [4], [5], [6], [7], [8], [9], [10], [11], [12], [13], [14], [15], [16], [17] for other socio-economic data.
• To understand the ethical practice of negotiation of professional fees within the approved standard and this can serve as basis for policy implementation by the appropriate professional and regulatory bodies.

## 1 Data

The data is a set of responses obtained from the administration of two different sets of questionnaires to Estate Valuers that deal in property valuation and their clients (commercial banks) within the Ikeja axis of Lagos State, Nigeria. The questionnaires were designed to solicit information on how much the professionals are willing to accept from their clients and also how much the clients are willing to pay assuming the property was financed through bank loan. Analysis of the data (responses from the questionnaires) can provide an insight on the various factors that can influence professional fees.

The list of all the supplementary data used in this article is summarized in Table 1.

**Table 1:** Supplementary materials.

Appendix	Data
A	Questionnaire administered to the clients
B	Questionnaire administered to the professionals
C	The response obtained from the clients in SPSS text file
D	The response obtained from the professionals in SPSS text file

## 2 Experimental design, materials and methods

**Fig. 1:**
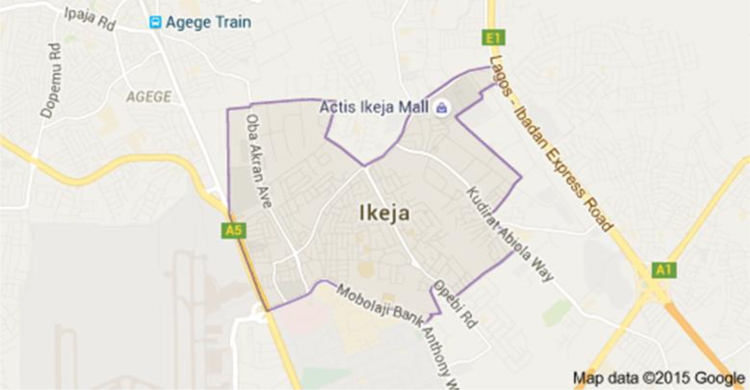
Map of Ikeja with the study area marked out. *Source:* Map – Google images/ maps [32].

The Estate Surveyors and Valuers Registration Board of Nigeria (ESVARBON) is a body that is statutorily responsible for the regulation of compensations paid by clients to professionals in the Nigerian Institution of Estate Surveyors and Valuers (NIESV). The compensations are in the form of scale upon which the agreed professional fees must be charged. However, the socio-economic realities in Nigeria have necessitated clients to negotiate the charges offered to them by the professionals. It should be noted that mortgage valuation is key to determination of professional fee. Surveys are very vital in understanding and predicting key population characteristics [18], [19], [20], [21], [22], [23], [24], [25], [26], [27], [28], [29], [30], [31]. In this case, Ikeja, Lagos, Nigeria was chosen for the research and the study area is indicated in Fig. 1.

The sampling frame is summarized in Table 2.

**Table 2:** The sampling frame.

Respondents	Sampling frame
Professionals	82 Registered real estate firms according to the directory of (NIESV) [33]
Clients	55 branches of commercial banks in the study area [34]

The sample size is estimated as a percentage of the sample frame using the formula;

$$n=\frac{z^{2}.p.q.N}{e^{2}\left(N-1\right)+z^{2}.p.q}$$

where:

*n* = sample size
*e* = permissible error
*p* = sample proportion
*q* = 1-sample proportion, that is, 1− *p*
*N*= sample population or sample frame.

This research adopted a confidence level of 95%, a sample proportion of 0.05, an allowable error of within ±5% of the true prevalence, with the sample frame for registered surveying firms as 82, and the sample frame for commercial banks as 55. The corresponding sample sizes will be calculated using the formula above. The sample size for the registered estate firms is given as:

$$\begin{array}{c} n=\frac{{\left(1.96\right)}^{2}.\left(0.05\times 0.95\times 82\right)}{{\left(0.05\right)}^{2}.\left(82-1\right)\;+\;{\left(1.96\right)}^{2}.\left(0.05\times 0.95\right)}\\ n=\frac{\left(3.4816\right).\left(3.895\right)}{\left(0.0025\right).\left(81\right)\;+\;\left(3.4816\right).\left(0.0475\right)}\\ n=\frac{13.5608}{0.2025\;+\;0.1825}\\ n=\frac{13.5608}{0.385}\\ n=35.22\\ n\approx 35 \end{array}$$

Thus a total of 35 registered Estate Surveying and Valuation Firms in the study area have been used as sample size, this represents about 42% of the total number of Estate Surveying and Valuation Firms within the sample frame.

The sample size for the commercial banks in the study area is calculated as:

$$\begin{array}{c} n=\frac{{\left(1.96\right)}^{2}.\left(0.05\times 0.95\times 55\right)}{{\left(0.05\right)}^{2}\left(55-1\right)\;+\;{\left(1.96\right)}^{2}.\left(0.05\times 0.95\right)}\\ n=\frac{\left(3.4816\right).\left(2.6125\right)}{\left(0.0025\right)\left(54\right)\;+\;\left(3.4816\right)\left(0.0475\right)}\\ n=\frac{9.0957}{0.135\;+\;0.1825}\\ n=\frac{9.0957}{0.3175}\\ n=28.6\\ n\approx 29 \end{array}$$

Thus a total of 29 commercial banks in the study area were used as the sample size; this represents about 53% of the total number of Estate Surveying and Valuation Firms within the sample frame that deal in property valuation.

Simple random sampling was used to administer the questionnaires to each group of respondents. The questionnaires were administered in English and the fees in Nigerian currency (Naira). The fees allowable are within the approved scale. Other factors bordering on ethics of the profession were included in the questionnaires.

Details on the studied population can be accessed in [33], [34].
