# Supplementary material for: Survey data on factors affecting negotiation of professional fees between Estate Valuers and their clients when the mortgage is financed by bank loan: A case study of mortgage valuations in Ikeja, Lagos State, Nigeria
Source: Data Brief. 2017 May 1;12:447–52. doi: 10.1016/j.dib.2017.04.047 (PMC5424955; doi:10.1016/j.dib.2017.04.047)
Supplement: Supplementary file 1 — Supplementary material [file mmc1.pdf]

COVENANT UNIVERSITY

COLLEGE OF SCIENCE AND TECHNOLOGY

DEPARTMENT OF MATHEMATICS

CANAANLAND, KM 10, IDIROKO ROAD
P.M.B 1023, OTA, OGUN STATE, NIGERIA
www.covenantuniversity.edu.ng, mat.covenantuniversity.edu.ng

EXTERNAL MEMO

To: Editor, Data in Brief
From: Corresponding Author
Date: 14th April, 2017
Subject: **Conflict of Interest**

I thereby declare the absence of any conflict of interest among the authors.

The authors have read the final draft and unanimously agreed that the paper be sent for review.

Hilary I. Okagbue
Department of Mathematics, Covenant University, Ota.
Google Scholar: Hilary Izuchukwu Okagbue
Research Gate: Hilary Okagbue
Live DNA: 234.14453
Scopus ID: 56438006100
ORCID: 0000-0002-3779-9763
