# Supplementary material for: Survey data on factors affecting negotiation of professional fees between Estate Valuers and their clients when the mortgage is financed by bank loan: A case study of mortgage valuations in Ikeja, Lagos State, Nigeria
Source: Data Brief. 2017 May 1;12:447–52. doi: 10.1016/j.dib.2017.04.047 (PMC5424955; doi:10.1016/j.dib.2017.04.047)
Supplement: Supplementary file 2 — Supplementary material [file mmc2.docx]

**
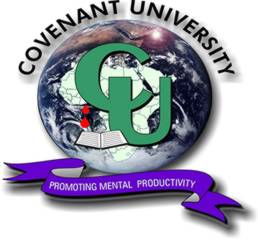
** COVENANT UNIVERSITY

OTA OGUN STATE

DEPARTMENT OF ESTATE MANAGEMENT

QUESTIONNAIRE

(For clients of mortgage valuation)

Dear Sir/Madam,

This questionnaire is designed to obtain information about your professional practice of negotiation with the Estate Valuers.

Please note that all information solicited for shall be treated with strict confidentiality and for research purpose only.

Thank you for your anticipated co-operation and prompt response.

**SECTION A: BASIC DATA**

1. Gender: (a) Male ( ) (b) Female ( ).
2. Age: (a) 18-28 (b) 29-38 (c) 39-49 (d) 50 and above.
3. Occupation/ Profession …………………….
4. Level of Educational status: (a) B.Sc. & HND holder (b) B.Sc. holder (c) HND holder (d) M.Sc. (e) PhD. (HND is Higher National Diploma)

**SECTION B**

Please indicate if you agree or disagree with the following items. Each represents a commonly held opinion and there is no right or wrong answers. The researcher is interested in your reaction to such matters of opinion. Rate your reaction to each statement by ticking the appropriate options which are inclusive of the following:

- Strongly agree
- Agree
- Undecided
- Strongly disagree
- Disagree

1. Do you think clients personal knowledge of Estate Valuers influence professional fees for mortgage valuation assignments?

| Strongly agree | Agree | Undecided | Disagree | Strongly Disagree |
| --- | --- | --- | --- | --- |
|  |  |  |  |  |

1. Do you think clients disposition influence professional fees for mortgage valuation assignments?

| Strongly agree | Agree | Undecided | Disagree | Strongly Disagree |
| --- | --- | --- | --- | --- |
|  |  |  |  |  |

1. Do you think that paying at shorter time reduces the professional fees charged by Estate Valuers?

| Strongly agree | Agree | Undecided | Disagree | Strongly Disagree |
| --- | --- | --- | --- | --- |
|  |  |  |  |  |

1. Do you think that the act of negotiation of professional fees with your Estate Valuer is always beneficial to you?

| Strongly agree | Agree | Undecided | Disagree | Strongly Disagree |
| --- | --- | --- | --- | --- |
|  |  |  |  |  |

1. Do you think that there should concessions for first time clients of estate valuation?

| Strongly agree | Agree | Undecided | Disagree | Strongly Disagree |
| --- | --- | --- | --- | --- |
|  |  |  |  |  |

1. Do you think that there should concessions for the size of clients in estate valuation?

| Strongly agree | Agree | Undecided | Disagree | Strongly Disagree |
| --- | --- | --- | --- | --- |
|  |  |  |  |  |

1. Do you think that your interests are adequately represented in negotiation with your Estate Valuer?

| Strongly agree | Agree | Undecided | Disagree | Strongly Disagree |
| --- | --- | --- | --- | --- |
|  |  |  |  |  |

1. Do you feel cheated negotiating professional fees with your Estate Valuer without a third party?

| Strongly agree | Agree | Undecided | Disagree | Strongly Disagree |
| --- | --- | --- | --- | --- |
|  |  |  |  |  |

1. As a client, does higher qualifications of the Estate Valuers justify the high professional fees charged by them?

| Strongly agree | Agree | Undecided | Disagree | Strongly Disagree |
| --- | --- | --- | --- | --- |
|  |  |  |  |  |

1. As a client, does the age and experience of the Estate Valuers justify the high professional fees charged by them?

| Strongly agree | Agree | Undecided | Disagree | Strongly Disagree |
| --- | --- | --- | --- | --- |
|  |  |  |  |  |

1. Do you think that the gender of the client helps in negotiation of professional fees charged by them?

| Strongly agree | Agree | Undecided | Disagree | Strongly Disagree |
| --- | --- | --- | --- | --- |
|  |  |  |  |  |

1. Do you think that the gender of the Estate Valuer plays an important role in negotiation of professional fees charged by them?

| Strongly agree | Agree | Undecided | Disagree | Strongly Disagree |
| --- | --- | --- | --- | --- |
|  |  |  |  |  |

1. Do you think that economic situation in the country determines the mode of negotiation of professional fees?

| Strongly agree | Agree | Undecided | Disagree | Strongly Disagree |
| --- | --- | --- | --- | --- |
|  |  |  |  |  |

1. Do you think that you can trust the judgment of a subordinate of the Estate Valuer?

| Strongly agree | Agree | Undecided | Disagree | Strongly Disagree |
| --- | --- | --- | --- | --- |
|  |  |  |  |  |

1. Do you think that the professional charges on mortgage valuation should be based on the value of the property alone?

| Strongly agree | Agree | Undecided | Disagree | Strongly Disagree |
| --- | --- | --- | --- | --- |
|  |  |  |  |  |

1. Do you think that the professional charges on mortgage valuation should be based on the value of the property and the loan sought for?

| Strongly agree | Agree | Undecided | Disagree | Strongly Disagree |
| --- | --- | --- | --- | --- |
|  |  |  |  |  |

1. What percentage of posted prices is slashed during the negotiation of professional fees on mortgage valuation assignments? (Please indicate your answer by ticking the appropriate box); 10% [ ] 20% [ ] 35% [ ] others (please specify)………..
2. Assuming, the fees for work done in respect of mortgage valuation, were based on the percentage of the property being valued; what percentage of the property should be charged for this purpose?

2.5% [ ] 5% [ ] 7.5% [ ] 10% [ ]

1. Assuming a property is valued at 100 million naira, how much would you satisfactory pay as fees for the work done in respect of mortgage valuation; assuming a loan is intended to be taken on the worth of the property, to the tune of (please indicate your answer in the appropriate spaces ):

50 Million naira ………………….

40 million naira ………………….

30 million naira ………………….

20 million naira ………………….

10 million naira ………………….

5 million naira ………………….

2 million naira ………………….

1 million naira ………………….

500 thousand naira ………………….
