# Supplementary material for: Survey data on factors affecting negotiation of professional fees between Estate Valuers and their clients when the mortgage is financed by bank loan: A case study of mortgage valuations in Ikeja, Lagos State, Nigeria
Source: Data Brief. 2017 May 1;12:447–52. doi: 10.1016/j.dib.2017.04.047 (PMC5424955; doi:10.1016/j.dib.2017.04.047)
Supplement: Supplementary file 3 — Supplementary material [file mmc3.docx]

**
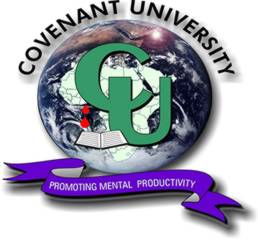
**COVENANT UNIVERSITY

OTA OGUN STATE

DEPARTMENTOF ESTATE MANAGEMENT

QUESTIONNAIRE

(For Estate Surveyors and Valuers)

Dear Sir/Madam,

This questionnaire is designed to obtain the information on the negotiation between you and your clients when the mortgage is financed through bank loans.

Please note that all information solicited for shall be treated with strict confidentiality and for research purpose only.

Thank you for your anticipated co-operation and prompt response.

**SECTION A: BASIC DATA**

1. Gender: (a) Male ( ) (b) Female ( ).
2. Age: (a) 18 -25 (b) 26-36 (c) 37-46 (d) and above.
3. Level of educational status: (a) B.Sc. & HND holder (b) B.Sc. holder (c) HND holder (d) M.Sc. (e) Ph.D.
4. Work experience? (a) 0-5 yrs (b) 6-10yrs (c) 11-20yrs (d) 21 and above
5. Professional qualification: (a) student (b) probationer (c) ANIVS (d) RSV (e) others

*Student implies practicing Estate Valuer with experience and in the process of passing the professional exams.

**Probationer is any practicing Estate Valuer between the period of passing the professional exams and certification.

**SECTION B: Ethical disposition of Estate surveyors to real estate practice**

Please indicate if you agree or disagree with the following items. Each represents a commonly held opinion and there is no right or wrong answers. The researcher is interested in your reaction to such matters of opinion. Rate your reaction to each statement by ticking the appropriate options which are inclusive of the following:

- Strongly agree
- Agree
- Undecided
- Strongly disagree
- Disagree

1. Professionals should make certain that their actions never intentionally cause harm to a client, even to a small degree.

| Strongly agree | Agree | Undecided | Disagree | Strongly Disagree |
| --- | --- | --- | --- | --- |
|  |  |  |  |  |

1. The existence of potential harm to clients is always wrong, irrespective of the benefits to be gained.

| Strongly agree | Agree | Undecided | Disagree | Strongly Disagree |
| --- | --- | --- | --- | --- |
|  |  |  |  |  |

1. A professional should not perform an action which might in any way threaten the dignity and welfare of a client.

| Strongly agree | Agree | Undecided | Disagree | Strongly Disagree |
| --- | --- | --- | --- | --- |
|  |  |  |  |  |

1. Client satisfaction should be the most important concern of any professional.

| Strongly agree | Agree | Undecided | Disagree | Strongly Disagree |
| --- | --- | --- | --- | --- |
|  |  |  |  |  |

1. Moral behaviors of professionals are actions that closely match the ideals of an established professional code of conduct.

| Strongly agree | Agree | Undecided | Disagree | Strongly Disagree |
| --- | --- | --- | --- | --- |
|  |  |  |  |  |

1. There are no ethical principles that are so important that they should be part of any code of ethics.

| Strongly agree | Agree | Undecided | Disagree | Strongly Disagree |
| --- | --- | --- | --- | --- |
|  |  |  |  |  |

1. What is considered professionally ethical varies from one profession to another.

| Strongly agree | Agree | Undecided | Disagree | Strongly Disagree |
| --- | --- | --- | --- | --- |
|  |  |  |  |  |

1. Rigidly codifying an ethical position that prevents certain types of actions for professionals could stand in the way of better human relations in dong business.

| Strongly agree | Agree | Undecided | Disagree | Strongly Disagree |
| --- | --- | --- | --- | --- |
|  |  |  |  |  |

1. Questions of what is ethical for a profession can never be resolved since what is moral or immoral is up to the individual professional.

| Strongly agree | Agree | Undecided | Disagree | Strongly Disagree |
| --- | --- | --- | --- | --- |
|  |  |  |  |  |

1. Ethical considerations in interpersonal relationships are so complex that professionals should be allowed to formulate their own individual codes.

| Strongly agree | Agree | Undecided | Disagree | Strongly Disagree |
| --- | --- | --- | --- | --- |
|  |  |  |  |  |

**SECTION C: questions on fees negotiation**

1. Does higher professional qualification of Estate Valuers signify higher professional fees charged by Estate Valuers?

| Strongly agree | Agree | Undecided | Disagree | Strongly Disagree |
| --- | --- | --- | --- | --- |
|  |  |  |  |  |

1. Does the experience of Estate Valuer affect the Valuer's negotiation of professional fees?

| Strongly agree | Agree | Undecided | Disagree | Strongly Disagree |
| --- | --- | --- | --- | --- |
|  |  |  |  |  |

1. Are male Estate Valuers more disposed to negotiating professional fees than their female colleagues are?

| Strongly agree | Agree | Undecided | Disagree | Strongly Disagree |
| --- | --- | --- | --- | --- |
|  |  |  |  |  |

1. Are older Estate Valuers in better position than their younger colleagues to negotiate professional fees?

| Strongly agree | Agree | Undecided | Disagree | Strongly Disagree |
| --- | --- | --- | --- | --- |
|  |  |  |  |  |

1. Are certified Estate Valuers in better position than those that are yet to be certified to negotiate appropriate professional fees?

| Strongly agree | Agree | Undecided | Disagree | Strongly Disagree |
| --- | --- | --- | --- | --- |
|  |  |  |  |  |

1. Do you think that there should be consideration in negotiation fees for first time clients?

| Strongly agree | Agree | Undecided | Disagree | Strongly Disagree |
| --- | --- | --- | --- | --- |
|  |  |  |  |  |

1. Do you think that there should be consideration in negotiation fees for colleagues, family members, friends, seasoned customers, Pastors, Iman, charity organizations, government officials?

| Strongly agree | Agree | Undecided | Disagree | Strongly Disagree |
| --- | --- | --- | --- | --- |
|  |  |  |  |  |

1. Does the size of the client determine the professional fees charged by Estate Valuers?

| Strongly agree | Agree | Undecided | Disagree | Strongly Disagree |
| --- | --- | --- | --- | --- |
|  |  |  |  |  |

1. Does the economic situation determine the professional fees charged by Estate Valuers?

| Strongly agree | Agree | Undecided | Disagree | Strongly Disagree |
| --- | --- | --- | --- | --- |
|  |  |  |  |  |

1. Do male Estate Valuers charge higher professional fees than their female colleagues?

| Strongly agree | Agree | Undecided | Disagree | Strongly Disagree |
| --- | --- | --- | --- | --- |
|  |  |  |  |  |

1. Do you think that negotiation between the professional and client can be delegated to subordinate?

| Strongly agree | Agree | Undecided | Disagree | Strongly Disagree |
| --- | --- | --- | --- | --- |
|  |  |  |  |  |

1. Do you think that third parties can be involved in negotiating professional fees between you and your client?

| Strongly agree | Agree | Undecided | Disagree | Strongly Disagree |
| --- | --- | --- | --- | --- |
|  |  |  |  |  |

1. Does the personal disposition of the Estate Valuer determine the professional fees charged by estate valuers?

| Strongly agree | Agree | Undecided | Disagree | Strongly Disagree |
| --- | --- | --- | --- | --- |
|  |  |  |  |  |

1. Does the operation cost and tax determine the professional fees charged by Estate Valuers?

| Strongly agree | Agree | Undecided | Disagree | Strongly Disagree |
| --- | --- | --- | --- | --- |
|  |  |  |  |  |

1. Does the Corporate governance from professional regulatory bodies determine the professional fees charged by Estate Valuers?

| Strongly agree | Agree | Undecided | Disagree | Strongly Disagree |
| --- | --- | --- | --- | --- |
|  |  |  |  |  |

1. Do you think that the professional charges on mortgage valuation should be based on the value of the property alone?

| Strongly agree | Agree | Undecided | Disagree | Strongly Disagree |
| --- | --- | --- | --- | --- |
|  |  |  |  |  |

1. Do you think that the professional charges on mortgage valuation should be based on the value of the property and the loan sought for?

| Strongly agree | Agree | Undecided | Disagree | Strongly Disagree |
| --- | --- | --- | --- | --- |
|  |  |  |  |  |

1. What percentage of posted prices is slashed during the negotiation of professional fees on mortgage valuation assignments? (Please indicate your answer by ticking the appropriate box); 10% [ ] 20% [ ] 35% [ ] others (please specify)………..
2. What is the minimum, you would charge as an estate surveyor and valuer, whereby a client engages your services for a mortgage valuation assignment, regardless of the scale and scope of the assignment? …………………
3. Assuming, the fees for work done in respect of mortgage valuation, were based on the percentage of the property being valued; what percentage of the property should be charged for this purpose?

2.5% [ ] 5% [ ] 7.5% [ ] 10% [ ]

1. Assuming a property is valued at 100 million naira, how much would you satisfactory charge as fees for the work done in respect of mortgage valuation ; assuming a loan is intended to be taken on the worth of the property, to the tune of:

50 Million naira ………………….

40 million naira ………………….

30 million naira ………………….

20 million naira ………………….

10 million naira ………………….

5 million naira ………………….

2 million naira ………………….

1 million naira ………………….

500 thousand naira ………………….
